# Use of a Molecular Genetic Platform Technology to Produce Human Wnt Proteins Reveals Distinct Local and Distal Signaling Abilities

**DOI:** 10.1371/journal.pone.0058395

**Published:** 2013-03-13

**Authors:** Jennifer L. Green, Matthieu Bauer, Kyu Won Yum, Yao-Cheng Li, Miranda L. Cox, Karl Willert, Geoffrey M. Wahl

**Affiliations:** 1 The Salk Institute for Biological Studies, La Jolla, California, United States of America; 2 Department of Cellular and Molecular Medicine, University of California San Diego, La Jolla, California, United States of America; University of Massachusetts Medical, United States of America

## Abstract

Functional and mechanistic studies of Wnt signaling have been severely hindered by the inaccessibility of bioactive proteins. To overcome this long-standing barrier, we engineered and characterized a panel of Chinese hamster ovary (CHO) cell lines with inducible transgenes encoding tagged and un-tagged human *WNT1*, *WNT3A*, *WNT5A*, *WNT7A*, *WNT11*, *WNT16* or the soluble Wnt antagonist *Fzd8CRD*, all integrated into an identical genomic locus. Using a quantitative real-time bioluminescence assay, we show that cells expressing WNT1, 3A and 7A stimulate Wnt/beta-catenin reporter activity, while the other WNT expressing cell lines interfere with this activation. Additionally, in contrast to WNT3A, WNT1 only exhibits activity when cell-associated, and thus only signals to neighboring cells. The reporter assay also revealed a rapid decline of Wnt activity at 37°C, indicating that Wnt activity is highly labile. These engineered cell lines will reduce the cost of making and purifying Wnt proteins and serve as a continuous, reliable and regulatable source of Wnts to research laboratories around the world.

## Introduction

Wnt signaling pathways are among the most important and most complex described in developmental biology. There are nineteen Wnt ligands that signal via receptors of the Frizzled family (ten members), Lrp co-receptors (two members) and receptor tyrosine kinases Ryk (one member) and Ror (two members) [1]. In addition, there is an expanding repertoire of agonists, antagonists and co-factors, including R-spondins and their receptors Lgr4/5, which were recently found to associate with Frizzled and Lrp5/6 [2]–[4], and ZNRF3 [5] and RNF43 (Koo et al. 2012, Nature 488: 665–669), which encode transmembrane ubiquitin ligases that associate with the receptor complex and target Fzd for degradation. In the canonical Wnt signaling pathway, Wnt binding to Frizzled, a seven-pass transmembrane receptor, and Lrp5/6, a single-pass co-receptor, triggers a cascade of events resulting in accumulation and nuclear-translocation of the transcriptional activator beta-catenin. Alternative mechanisms of Wnt signaling, often referred to as ‘non-canonical,’ do not stimulate beta-catenin-mediated gene transcription, but rather trigger changes in cell morphology, motility and polarity [6]. These alternative mechanisms can involve the Ryk and/or Ror receptors and are often associated with repression of canonical Wnt signaling. While Wnts were historically divided into two classes, canonical and non-canonical, recent evidence suggests that canonical Wnts can be further sub-divided into multiple families based on their interaction with the Lrp co-receptor. For example, members of the Wnt1 class are predicted to bind to the first YWTD-type beta-propeller domain in the ectodomain of Lrp6, while members of the Wnt3a class are predicted to bind to the third Lrp6 YWTD-type beta-propeller domain [7], [8]. Whether non-canonical Wnts share similar or different downstream signaling mechanisms is presently unclear. In addition, it should be noted that certain non-canonical Wnts can activate beta-catenin in certain contexts [9]–[12].

Understanding the complexity of Wnt signaling is confounded by the intractable nature of Wnt proteins themselves; they are notoriously difficult to express, purify and maintain in a bioactive state. Wnts are modified post-translationally by glycosylation and acylation and have a tendency to be retained in the endoplasmic reticulum and the cell surface [13]. Covalently attached lipid moieties render Wnt proteins hydrophobic and likely contribute to their poor solubility and tendency to aggregate. The recently published high resolution structure of a Wnt protein in complex with the ligand binding domain of Frizzled, the Cysteine rich domain (CRD), shows that Wnt ‘grabs’ the CRD with a ‘thumb’ and ‘index finger’ (Janda et al. 2012). In this structure, the lipid moiety extends from the thumb and is critically important in receptor binding.

Most functional studies to date have focused on mouse Wnt3A (mWnt3A) and mouse Wnt5A as representative examples of canonical and non-canonical Wnts, respectively. The substantial efforts studying these Wnts do not imply a lack of importance of the others, but rather reflect the fact that these were the first Wnts to be purified [11], [14], thus enabling extensive experimentation. Cell lines secreting mWnt3A and mWnt5A are available to researchers through the ATCC. While a number of recombinant Wnt proteins are commercially available, their high costs frequently preclude large-scale experiments. In addition, these commercially available Wnts exhibit highly variable concentrations, activities and purities (K. Willert and M. Bauer, unpublished observations and [15]). Furthermore, although purified protein may be ideal for acute studies, it is not practical for longer-term studies as Wnt proteins lose activity quickly in culture media (this report and Derk ten Berge, personal communication). Transient transfection with Wnt expression vectors bypasses some of the issues associated with purified Wnt proteins, but raises concerns about the consequences of heterogeneous expression levels and interpretation of a population's phenotype based on effects of supra-physiological Wnt levels in a fraction of transfected cells.

Co-culture systems with cells that stably express Wnts provide a convenient alternative for generating continuous sources of active Wnts. It follows that a system in which production of bioactive Wnts is tunable would provide numerous experimental advantages. Although the commonly used mouse L cells can produce bioactive Wnt proteins, they are not generally preferred for protein production. Rather, CHO cells are the most widely used cell line for large-scale protein production in the biotechnology industry [16]. CHO cells reliably generate high protein yields, can be grown at high-density under chemically defined conditions and are adaptable to suspension culture, thus permitting large scale production in bioreactors [17].

Here, we describe a system that incorporates Cre recombinase mediated cassette exchange (RMCE) and inducible transgene expression to generate eleven new CHO cell lines expressing tagged and un-tagged human Wnt genes and the Wnt antagonist *Fzd8CRD*. We used Cre to deliver the Wnt expression transgenes into a floxed locus downstream of the *DHFR* locus [18]. This strategy ensured that the integrated Wnt transgene is present in a defined locus, enabling reproducible and consistent expression of different Wnt proteins between independently derived cell lines. Furthermore, this system was designed to provide Doxycycline (Dox)-inducible expression to enable tunable expression levels. We used this system to purify hWNT3A and hWNT5A proteins from conditioned media, and found that these proteins are active in a new real-time bioluminescence Wnt reporter assay. These cell lines should make functional studies of human Wnt systems more accessible, reliable and reproducible in research laboratories and reduce the cost of making and purifying Wnt proteins.

## Results

### Engineering Wnt-producing iCHO cell lines

**Figure 1:**
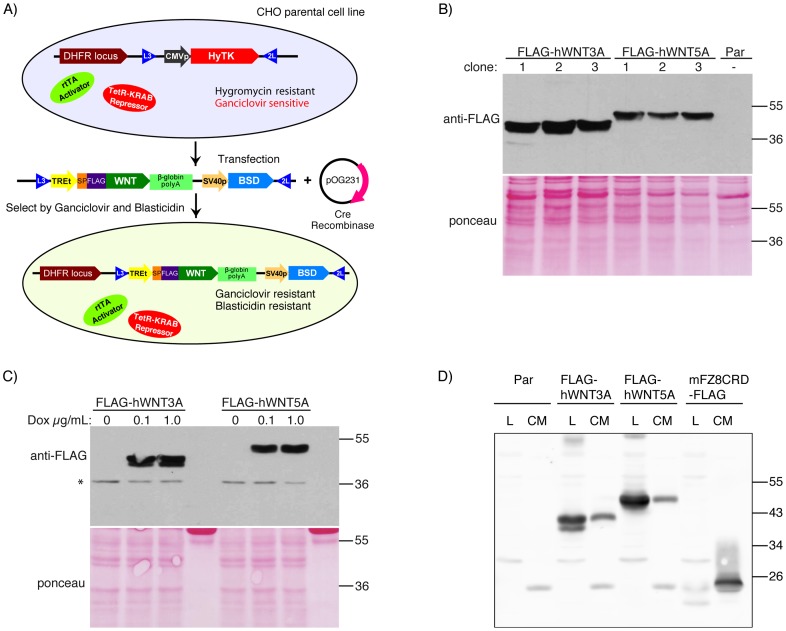
Generation of Wnt-expressing iCHO clones. A) Schematic of RMCE strategy. Parental CHO cells containing TetR-KRAB, rtTA and a genomic acceptor cassette, located downstream of the dihydrofolate reducatse (DHFR) locus, were co-transfected with a plasmid containing the incoming exchange cassette and a plasmid encoding Cre recombinase. Upon expression of Cre, the L3 and 2L recognition sequences in the genome are recombined with the L3 and 2L recognition sequences in the incoming exchange cassette. This results in excision of HyTK, thus rescuing Ganciclovir sensitivity, and insertion of the Wnt-expression cassette, which confers Blasticidin (BSD) resistance. B) Anti-FLAG western blot of cell lysates shows expression of FLAG-hWNT3A and FLAG-hWNT5A in three different iCHO clones. * Indicates non-specific band. Protein loading was visualized by Ponceau staining (bottom). C) Cells were treated with 0, 0.1 or 1.0 µg/mL Dox. Anti-FLAG western blot of cell lysates shows expression of FLAG-hWNT3A and FLAG-hWNT5A. Near maximal expression was achieved with 0.1 µg/mL Dox. D) iCHO cells were grown in the presence of 0.25 µg/mL Dox for three days at which point CM and cell lysates (L) were collected. Wnts and mFZ8CRD were immunoprecipitated from CM using anti-FLAG sepharose. While two species of FLAG-hWNT3A were visible in the cell lysate, only one form was visible in the CM, suggesting that the smaller species is not secreted.

We previously described a Dox-controlled transgene expression system in mammalian cells in which a transgene was efficiently targeted to a genomic locus [18]. The locus was selected to exhibit low basal expression, and to confer tightly-regulated Dox-inducible transgene expression. The parental CHO cell line contains a genomic acceptor cassette comprised of HyTK, which confers Hygromycin resistance and Ganciclovir sensitivity, flanked by the heterologous LoxP sites L3 and 2L (Fig. 1A). The parental line also contains the reverse tetracycline transactivator (rtTA) and the TetR-KRAB repressor integrated at a separate genomic location to confer Dox inducibility with minimal expression in the absence of Dox. Importantly, the integrated transgene exhibits reproducible levels of Dox-dependent induction in independently derived clones.

The average RMCE efficiency in these CHO cells was extremely high, with 80% of drug selected clones carrying a site-specific insertion [18]. More recently, we have found that false-positive RMCE clones can result from either loss of the HyTK cassette, or silencing of the TK gene (data not shown), both of which confer Ganciclovir resistance. We developed the following strategy to minimize emergence of non-RMCE induced Ganciclovir resistant clones. We introduced a Blasticidin drug resistance gene (BSD) into the donor exchange vector and optimized the RMCE selection scheme with two rounds of drug selection: first, Ganciclovir treatment selects for the absence of the HyTK cassette; second, Blasticidin treatment selects for the presence of the transgene cassette (Fig. S1A). This improved RMCE selection strategy results in the generation of targeted clones with greater than 99% fidelity (Fig. S1B).

To express human *WNT* genes, the incoming exchange vector contains human WNT cDNA under transcriptional control of the TRE-tight promoter (seven tetracycline response elements combined with the TATA box from the minimal CMV promoter), which is inducible by Tetracycline or its analog Dox. We engineered the human *WNT3A* gene to insert a sequence encoding a single FLAG tag. The resulting FLAG-tagged hWNT3A protein carries the FLAG epitope at its N-terminus following signal sequence cleavage. To generate a FLAG-tagged WNT5A protein, we engineered the *WNT5A* gene to carry the sequence encoding the WNT3A signal sequence followed by FLAG in the place of the WNT5A signal sequence. The soluble Wnt inhibitor mFzd8CRD [19], [20] contains a FLAG tag at the C-terminus. The incoming exchange vector was transfected into the parental CHO cell line along with a Cre-recombinase expression vector. After sequential rounds of selection with Ganciclovir and BSD, clones were isolated and transgene expression was confirmed by immuno-blotting (Fig. 1B). Using this strategy, we rapidly generated multiple clones of inducible CHO lines (iCHO) with undetectable background Wnt expression and similar levels of Wnt expression upon Dox treatment (Fig. 1C). Anti-FLAG immunoprecipitation confirmed the presence of WNT3A, WNT5A and mFzd8CRD in conditioned media (CM, Fig. 1D).

### iCHO cells express active human Wnt proteins in a tunable manner

**Figure 2:**
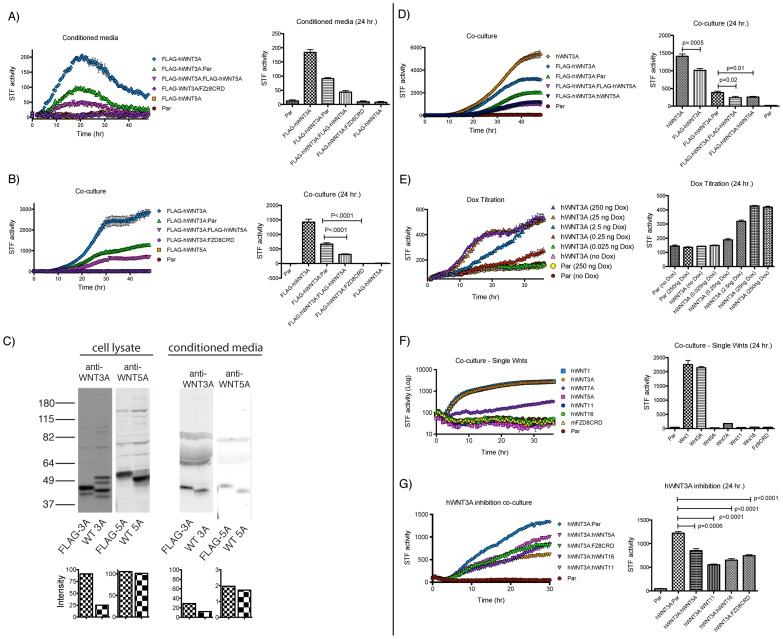
Activity of iCHO-produced Wnt Proteins. The graphs on the left depict induction of SUPERTOPFLASH (STF) activity in real-time bioluminescence monitoring assays. Values from the 24 hour timepoint were extracted for statistical analysis and are presented in the charts on the right.A) CM was collected from iCHO cells grown in the presence of Dox and 100 µL was added to reporter cells (mouse L + STF) in a 96 well plate. The volume of CM was kept constant such that ‘FLAG-hWNT3A:Par’ contained 50 µL FLAG-hWNT3A CM and 50 µL CM from parental CHO cells. Thus, ‘FLAG-hWNT3A:FLAG-hWNT5A’ CM and ‘FLAG-hWNT3A:Par’ each have an equal amount of FLAG-hWNT3A CM. Arrow points to FLAG-hWNT1 in CM. B, D–G) 293A-STF cells and iCHO cells were seeded together in a 96 well plate in the presence of Dox. Accumulation of luciferase activity is delayed compared to CM, presumably because it takes some time for the iCHO cells to produce Wnt following Dox stimulation. The total number of iCHO cells per well was kept constant such that ‘FLAG-hWNT3A’ contained 100% FLAG-hWNT3A cells and ‘FLAG-hWNT3A:Par’ contained 50% FLAG-hWNT3A cells and 50% Parental CHO cells. C) Anti-WNT3A and anti-WNT5A western blots comparing the levels of tagged and untagged WNTs in cell lysates and conditioned media. Quantification is shown below, FLAG staining was normalized to non-specific bands.

The Super-TOPFLASH (STF) luciferase reporter assay is a well-established indicator of canonical Wnt activity [21], [22], such as Wnt3A. In contrast, non-canonical Wnt5A activity can be detected by its ability to inhibit Wnt3A-induced STF activity [6], [11]. Inhibition of canonical Wnt signaling by non-canonical Wnt signaling has been observed previously in several settings, including in Xenopus (Torres et al. 1996) and in mammalian cell culture (Ishitani et al. 1999 and 2003). Using the STF reporter, we evaluated the activity of iCHO-produced Wnts by real-time bioluminescence monitoring assays [23], whereby STF luciferase activity was measured in live cells over 24–48 hours. Treatment of mouse L cells that stably carry the STF reporter [11] with iCHO-produced FLAG-hWNT3A CM resulted in robust induction of luciferase activity that peaked ∼20 hours post treatment (Fig. 2A). This activity was inhibited ∼50% by FLAG-hWNT5A and ∼100% by mFzd8CRD-FLAG CM, indicating that iCHO cells secrete Wnts with the expected activities. In co-culture experiments, where iCHO cells and 293A-STF reporter cells were grown in the same well, Wnt activity was more sustained compared to stimulation by CM (Fig. 2B), suggesting that Wnts in the CM become depleted or inactivated over time (see below). The parental iCHO cell line failed to induce reporter expression under either condition, indicating low or absent endogenous expression of canonical Wnts by these cells.

We next generated iCHO cell lines expressing untagged versions of hWNT3A and hWNT5A to address whether the FLAG tag altered Wnt activity. Although FLAG-hWNT3A was more abundant than untagged hWNT3A in iCHO cell lysates and CM (Fig. 2C), iCHO-FLAG-hWNT3A cells induced a STF response that plateaued at close to 58% of that induced by iCHO- hWNT3A cells (Fig. 2D), indicating that the FLAG tag partially reduces the activity of WNT3A protein. In contrast, FLAG-hWNT5A and hWNT5A behaved similarly, as measured by antagonism of WNT3A activity (Fig. 2D) and were similarly expressed and secreted. This is consistent with a recent report that FLAG-mWnt5A retains activity [12].

As Wnts are morphogens that exert their effects on responding cells in a concentration dependent manner [24], an optimal system would enable modulation of Wnt expression levels. We therefore assessed the inducibility of *WNT3A* expression by treating iCHO-hWNT3A cells with varying Dox concentrations and measuring induction of the Wnt-dependent reporter in co-culture with 293A-STF cells. The results clearly show a Dox-dependent induction of Wnt reporter activity in this co-culture system (Fig. 2E). In agreement with immuno-blot analysis (see Fig. 1C), uninduced iCHO-hWNT3A (i.e. no Dox) and parental CHO cells caused virtually the same background luminescence levels when mixed with the Wnt-responsive 293-STF cells, indicating that this inducible system has little if any expression in the absence of inducer, and a very high signal to noise ratio upon Dox addition (Fig. 2E).

Non-canonical or beta-catenin independent Wnt signaling involves multiple potentially overlapping signaling pathways, including Wnt-Calcium signaling and Planar Cell Polarity. These pathways have been especially difficult to investigate in part because mWnt5A is the only non-canonical Wnt encoded by a Wnt-producing cell line available to the research community [25]. We generated *hWNT11* and *hWNT16* expressing iCHO cell lines to enable further study of beta-catenin-independent Wnt signaling mechanisms. Like WNT5A, neither WNT11 nor WNT16 induced STF in co-culture (Fig. 2F) and both inhibited WNT3A activity to a similar degree (Fig. 2G).

### Purification of bioactive human Wnt proteins

**Figure 3:**
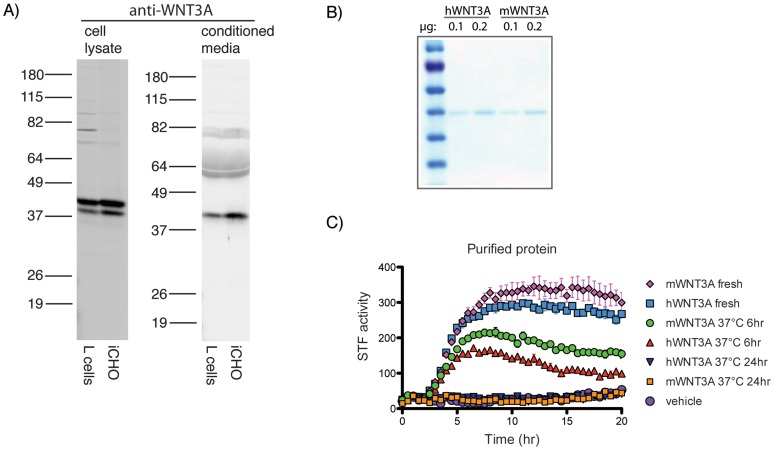
Wnt production, secretion and stability. A) Anti-WNT3A western blots comparing the levels of mWNT3A produced by L cells or iCHO cells in cell lysates and conditioned media. B) Coomassie stain of purified hWNT3A and mWNT3A separated by SDS-PAGE. C) 293A-STF cells were treated with 200 ng/mL purified hWNT3A or mWnt3A protein that was either fresh from 4°C, or pre-incubated at 37°C for 6 hr or 24 hr.

We adapted methods to purify mWnt3A from L cell CM [14], [26] to purify mWnt3A from iCHO CM and found iCHO cells to be a superior source of mWnt3A protein. iCHO cells produce and secrete more mWnt3A than L cells (Fig. 3A). In addition, collecting and filtering iCHO CM is more efficient because iCHO cells adhere even at high cell densities, which permits successive harvests of CM from expanded cell populations. In contrast, L cells detach at high densities and are present in the harvested CM, leading to slow filtration through 0.2 µm filters, a process required for the subsequent column purification (data not shown).

We successfully used the same method to purify human Wnts (hWNT3A and hWNT5A) from iCHO CM. Both purified hWNT3A (Fig. 3B, C) and hWNT5A (Fig. S2) displayed the expected activity. Purified hWNT3A from iCHO CM and mWnt3A from L cell CM migrated at the same position on an SDS-polyacrylamide gel (Fig. 3B). While this suggests that the two proteins share similar post-translational modifications, mWnt3A displayed slightly higher activity. Both mWnt3A and hWNT3A rapidly lost activity when pre-incubated in culture media at 37°C (Fig. 3C), suggesting that continuous production in a co-culture environment with iCHO cells may be advantageous when sustained Wnt signaling is required.

### Distinct Properties of Different Canonical Wnt proteins

Although Wnt3A is widely considered as representative of all canonical Wnts, it was recently reported that canonical Wnts can be subdivided into three groups based on their interaction with the LRP6 co-receptor (Ettenberg et al. 2010, Gong et al., 2010). We thus generated additional iCHO cell lines expressing *hWNT1* and *hWNT7A* to represent each of the other groups.

**Figure 4:**
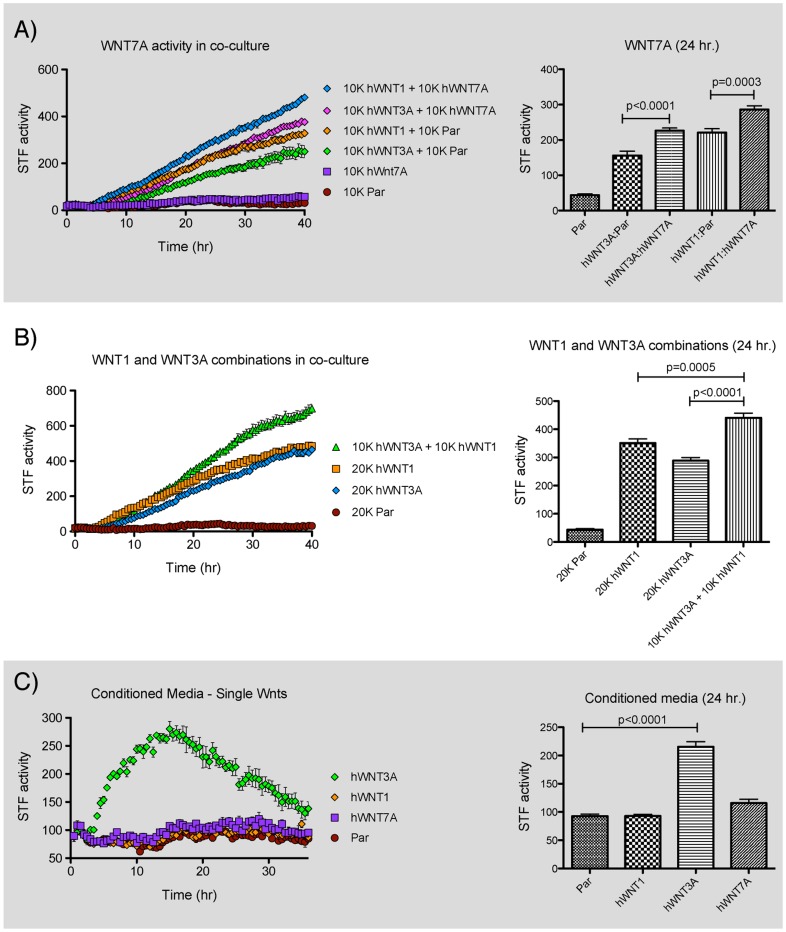
Different Wnt proteins display distinct activities. The graphs on the left depict activity in real-time bioluminescence monitoring assays. Values from the 24 hour timepoint were extracted for statistical analysis and are presented in the charts on the right.A–C) 293A-STF cells and CHO cells were seeded together in a 96 well plate in the presence of Dox, the total number of CHO cells per well was kept constant.

The canonical Wnts WNT3A and WNT1 robustly activated STF in co-culture assays (Fig. 2F). WNT7A alone exhibited weak and delayed induction of STF, but showed clear and reproducible enhancement of WNT3A and WNT1 activity in combinatorial assays in which the total number of iCHO-Wnt cells in each condition was constant (Fig. 4A). We also observed increased STF induction when WNT1 and WNT3A were introduced together, consistent with recent reports (Fig. 4B) (Ettenberg et al. 2010, Gong et al., 2010).

**Figure 5:**
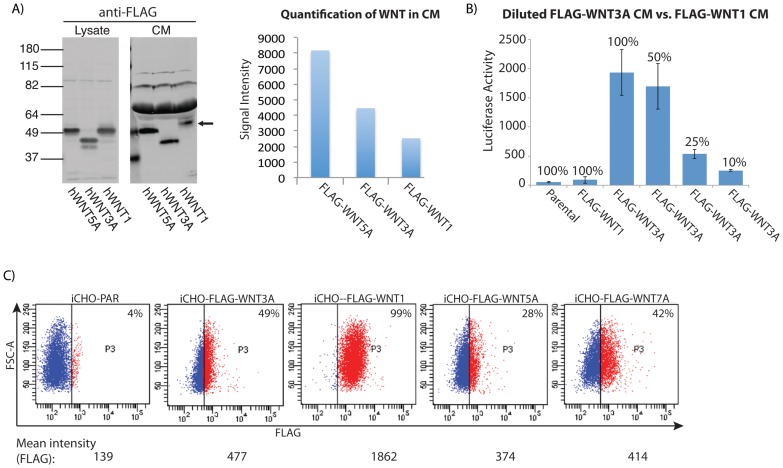
WNT1 is retained on the cell surface. A) Right - Western blot detecting the FLAG epitope comparing the amounts of hWNT1, hWNT3A and hWNT5A in cell lysates and conditioned media. Arrow points to hWNT1. Left - Quantification of relative WNT protein levels in CM. B) WNT3A CM was diluted to approach the WNT concentration of WNT1 CM (FLAG-WNT3A 50%), and then diluted further (FLAG-WNT3A 25%, FLAG-WNT3A 10%). In each case, WNT3A CM elicited a STF reporter response, while undiluted WNT1 CM did not. C) Flow cytometry analysis detecting the FLAG epitope comparing the amount of different WNTs on the surface of live iCHO cells following Dox induction.

WNT3A CM robustly activated STF, although the magnitude was lower than that observed with co-culture (Fig. 4C). WNT5A likewise had activity in CM (Fig. 2A). In contrast, while WNT1 was a potent inducer in co-culture, WNT1 CM failed to induce STF. This difference in activity could be due to reduced expression or secretion of WNT1 compared to the other Wnts. We thus generated iCHO cells expressing *FLAG-hWNT1* and used the common FLAG epitope to compare protein levels of FLAG-WNT1 to FLAG-WNT3A and FLAG-WNT5A in iCHO cell lysates and CM. Although all three Wnts were present in cell lysates at similar levels, less FLAG-WNT1 was present in the CM than either FLAG-WNT3A or 5A (Fig. 5A). Furthermore, the FLAG-WNT1 protein in CM migrated slower on an SDS-polyacrylamide gel than the corresponding protein in the cell lysate, suggesting that secreted WNT1 protein is more heavily modified than WNT3A and 5A. Of note, WNT1 contains four predicted glycosylation sites, all of which are modified in mWnt1 [27] while WNT3A contains only two [13]. We compared FLAG-WNT1 CM with dilutions of FLAG-WNT3A CM in the STF assay to determine whether the failure of WNT1 CM to induce STF resulted from lower protein concentration (Fig. 5B). Even at one-sixth the concentration of WNT1 CM, WNT3A CM still elicited an STF response. Thus in contrast to WNT3A, secreted and soluble WNT1 protein is not active when assayed in this way.

Lack of soluble WNT1 activity in CM raised the possibility that WNT1 may act more locally than WNT3A, which retains activity in CM. Localized function of WNT1 would require that signaling and responding cells be in close proximity, possibly requiring cell-to-cell contact, leading us to hypothesize that WNT1 may be preferentially retained on the surface of the producing cell, compared to WNT3A. Flow cytometry using the FLAG epitope revealed a greater signal on live FLAG-WNT1 iCHO cells than FLAG-WNT3A, FLAG-WNT5A or FLAG-WNT7A iCHO cells (Fig. 5C). The anti-FLAG signal was sensitive to trypsinization, confirming that FLAG-WNT protein is on the cell surface (Fig. S3).

**Figure 6:**
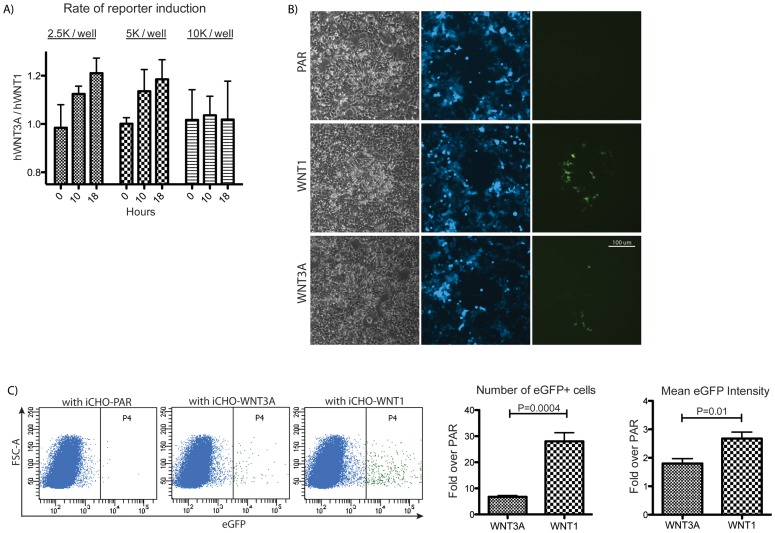
WNT1 acts locally. A) iCHO cells expressing untagged hWNT1 or hWNT3A were co-cultured with 293A-STF cells at varying cell densities. The ratio of hWNT3A-induced STF activity to hWNT1-induced STF activity is shown for three timepoints. The rate of reporter induction by WNT3A is higher than WNT1 at low cell densities, but not at high cell densities. B) The BSTG cell line was generated to distinguish iCHO cells (uncolored) from 293 reporter cells (blue). iCHO cells were seeded at 1000 cells per 10 cm plate and allowed to form colonies for 4 days prior to the addition of BSTG cells. Dox was added to plates after 24 hours to induce Wnt expression. Images were taken 48 hours later. iCHO colonies were located based on cell morphology and confirmed by corresponding gaps in the blue channel. The BSTG cell line is not clonal and cells exhibit varying levels of blue fluorescence. Unprocessed GFP images taken with identical exposure settings are shown, additional unprocessed images are shown in Fig. S6. C) Flow cytometry analysis of the cells used in co-culture imaging experiments. The plots show GFP expression of 50,000 live BSTG cells. The bar graphs depict the number of GFP+ cells in the ‘P4’ gate (left) and the geometric mean GFP intensity of those cells (right).

We next used several different approaches to assess the relative abilities of untagged WNT1 and WNT3A to activate Wnt signaling locally versus distantly. We reasoned that if soluble WNT3A is active and soluble WNT1 is not, then at lower cell densities, WNT3A should accumulate in the media over time, and induce reporter expression in responding cells at a greater rate than WNT1. At higher densities, when cell-to-cell contact is saturated, the rate of reporter induction should be similar between WNT1 and WNT3A. This is indeed what we observed (Fig. 6A). These results suggest that WNT1-mediated signaling requires that cells be in close proximity, while WNT3A can act at a distance.

As a second and more direct test of the hypothesis that WNT1 acts proximally, we determined whether WNT1 activity is localized to cells adjacent to a WNT1 source. We performed a co-culture imaging experiment where a few small colonies of iCHO cells expressing untagged Wnts were established and then overlaid with excess HEK293 cells carrying the SuperTOPeGFP reporter [28]. We engineered these cells to constitutively express a blue fluorescent protein (TagBFP) to distinguish them from the iCHO cells. We routinely observed a halo of bright GFP-positive BSTG (Blue SuperTOPeGFP) cells surrounding iCHO-WNT1 colonies forty-eight hours following Dox addition (Fig. 6B, Fig. S4). GFP-positive cells were also observed near iCHO-WNT3A colonies, however these cells were dimmer and scarcer than those seen with iCHO-WNT1 colonies. Induction of the SuperTOPeGFP reporter was not observed in cells that were not adjacent to an iCHO WNT source in either condition and was never observed on plates with parental CHO cells. As SuperTOPeGFP expression is dose sensitive (Fig. S5), these results suggest that iCHO-WNT1 cells induce Wnt signaling in their immediate neighbors, while WNT3A is diluted by diffusion into the media resulting in a lower level of induction near the source and fewer responding cells. These differences were quantified by flow cytometry analysis of the cells used in the above imaging analysis (Fig. 6C). In co-culture with iCHO-WNT1 cells, a greater number of BSTG cells expressed GFP and showed more intense signal compared to those co-cultured with iCHO-WNT3A. Consistent with the imaging analysis, where WNT responding cells were only found adjacent to the infrequent iCHO colonies, very few GFP-positive cells were detected in any condition by flow cytometry.

## Discussion

This report describes an improved and highly efficient RMCE-based system to produce proteins of biologic interest in mammalian cells. We provide proof-of-principle by applying this method to generate a family of cell lines that inducibly express human *WNT* genes or a Wnt antagonist. We chose the Wnt family of proteins to exemplify the robustness and versatility of this strategy because Wnt proteins are notoriously challenging to work with, difficult to obtain and because of their relevance to developmental and disease biology.

Others have recognized the inaccessibility of human Wnts as a critical barrier to overcome, especially as pathway modulators enter clinical trials. For example, a new depository of expression plasmids for all nineteen human Wnt genes has been made available [29]. While these plasmids encode *WNTs* controlled by the same expression vector, variability in copy number and integration site following transfection make it difficult to predict or control expression levels between *WNTs* using these plasmids. Importantly, this study showed that addition of three N-terminal FLAG tags or a single C-terminal V5 tag is incompatible with WNT activity. Wnt3a with a single C-terminal FLAG tag is similarly inactive (our unpublished observation). Thus, the single N-terminal FLAG tag used here, which only partially reduces WNT activity, may be the optimal way to add a common epitope for WNT detection.

We developed a kinetic Wnt reporter assay to characterize the biological activity of the proteins generated by each cell line. Using this assay, we found variability in the peak of Wnt-induced luciferase activity measured for different cell lines responding to the same Wnt. For example, maximal response to WNT3A of an L-cell STF reporter line occurred at ∼20 hours (Fig. 2A), while a 293A-based reporter system occurred closer to 15 hours post treatment (Fig. 4C). Although most current studies measure Wnt signaling by endpoint STF luciferase assays, our method shows that critical effects can be missed if a time point is chosen in advance of, or following, the peak. Thus, the kinetic assay described here provides a more comprehensive and reliable means of evaluating Wnt activity.

We noted that while co-culture with WNT3A-producing cells induced a sustained STF response, CM produced a response that peaked and then declined within the assay period. This could be due to the Wnt in the CM being depleted, to receptor turnover in the responding cells, or to loss of Wnt protein activity over time, for example by degradation. By pre-incubating the protein at 37°C prior to treating the cells, we demonstrated that WNT3A loses much of its activity after 6 hours and is completely inactive after 24 hours. Thus, long-term experiments with Wnt proteins would require regular reintroduction of fresh protein to maintain signaling. This presents a technical challenge because purified Wnt protein is stored in a high detergent buffer and accumulation of this buffer can be toxic to cells. Furthermore, periodic replenishment may produce non-physiological activity spikes that do not model *in vivo* signaling processes. Where possible, co-culture can be an easy and affordable alternative to maintain a steady supply of Wnt, or other biologically active proteins or peptides, in the media.

Among the non-canonical Wnts, we found that WNT5A, WNT11 and WNT16 each inhibited WNT3A activity to a similar degree (Fig. 2G). The ability of WNT5A and WNT11 to inhibit canonical Wnt signaling is well established [6], [11], [30]. However, to our knowledge, a similar function for WNT16 has not been previously described. Given recent evidence that WNT16 plays a role in the specification of hematopoietic stem cells and in human leukemia, it is important to elucidate such mechanistic properties of Wnt16 signaling [31]–[34]. It should be noted that our observations of ‘non-canonical’ Wnt proteins (WNT5A, WNT11, WNT16) and their ability to block Wnt/beta-catenin signaling is consistent with a model of ligand-receptor competition where the non-canonical Wnt protein competes for receptor binding with WNT3A but fails to engage the receptor in a manner that leads to signal transduction. This alternative model is particularly appealing in light of the recent demonstration that WNT5A inhibits beta-catenin signaling in mouse cells lacking ROR1 and ROR2, the receptors proposed to transduce a signal that antagonizes beta-catenin signaling [35].

Our studies reveal important distinctions between individual Wnt proteins. While both WNT1 and WNT3A displayed similar activity in co-culture, only WNT3A exhibited activity in CM. This is consistent with previous reports using fibroblasts expressing mWnt1 where it was shown that the majority of this protein was associated with the extracellular matrix, and that little or none was detectable in the conditioned media [36]. This phenomenon has been reported for other Wnt1-expressing cell types as well [37], [38], but was never compared to other Wnts in the same system to show whether this property is specific to Wnt1. This prior work also indicates that failure to produce long-range acting WNT1 is not specific to CHO cells. Using FLAG-tagged Wnt proteins, we showed that the WNT1 level in CM was lower than that of WNT3A, but was clearly present. As the STF assay is sufficiently sensitive to detect activity from a comparable or lower amount of WNT3A, we infer that the WNT1 protein present in CM is inactive. Using flow cytometry we further showed that WNT1 is retained on the cell surface to a greater extent than WNT3A or the other Wnts tested. This result and our finding that the rate of WNT1-induced signaling is dependent on cell density are consistent with WNT1 exhibiting its activity over a limited distance. Together, these data suggest that unlike WNT3A, WNT1 is only active when cell associated, not after release into the media. In support of this model, co-culture imaging experiments showed a halo of WNT-responding BSTG cells surrounding iCHO-WNT1 colonies. This result is reminiscent of the morphological transformation of rings of mouse C57MG mammary cells surrounding colonies of *Wnt1*-overexpressing Rat-2 cells [39]. Surprisingly, we did not observe GFP expression in all BSTG cells upon co-culture with iCHO WNT3A cells, but instead observed very few cells with activated Wnt signaling. One explanation is that the low amount of WNT3A present under conditions where iCHO-WNT3A colonies are small and sparse and release into the media rather than concentration on the surface, results in dilution by diffusion into the media and an inability to reach the necessary concentration threshold to activate the pathway in all cells. These differences in cell association and soluble activity could reflect different biological functions whereby WNT1 is effective at proximal intercellular communication, possibly requiring direct cell:cell contact, while WNT3A behaves more like a classical morphogen able to act over a distance. This distinction could be important during developmental morphogenesis or stem cell-niche interactions when the spatial organization of cells is crucial.

It has been proposed that long-range and short-range Wnt signaling are executed by different forms of Wnt protein, which are produced by distinct secretory mechanisms [13]. Long- versus short-range signaling could be determined by sorting of secretory vesicles to different membrane subdomains, where WNTs may encounter distinct co-factors, such as lipoprotein particles or the secreted WNT interacting molecule Swim that regulate WNT's distribution in the extracellular space [40]. While WNT1 and WNT3A are similar in length, molecular weight and in the number of cysteines they possess, WNT1 has twice as many potential N-glycosylation sites as WNT3A [13]. N-glycosylation is a sorting signal for other secreted proteins [41]), Although the precise function of N-glycosylation in Wnt maturation and processing is unclear, the system presented here is ideally suited to determine whether N-glycosylation is involved in sorting Wnt proteins along specific secretory routes and whether this contributes to long- versus short –range activity.

Tiki1 and Tiki2 were recently identified as potential proteases that efficiently inactivate Wnts by cleaving eight amino-terminal residues (following the signal sequence), which changes the hydrophobicity and disrupts receptor binding [42]. The robust activity exhibited by iCHO-produced Wnts makes it unlikely that Tiki is functioning as a posttranslational Wnt-inactivator in this system. Also, Wnts processed by Tiki exhibit faster electrophoretic mobility, yet Wnt1 present in the CM of iCHO cells migrates slower than Wnt1 present in the cell lysate suggesting that posttranslational inactivation by Tiki is probably not responsible for the lack of Wnt1 activity in iCHO CM. Furthermore, Wnt3A secreted by iCHO cells migrates at the same position as Wnt3A secreted by L cells (Figure 3B), which is not apparently cleaved by Tiki [42]. Finally, only one band is detected in CM from iCHO-FLAG-hWNT3A cells, using an antibody against WNT3A, and this protein is similarly detected with anti-FLAG, indicating that the N-terminus is not cleaved. Thus, lack of endogenous Tiki activity makes this system well suited to study how Tiki processes different Wnt proteins.

The eleven Wnt-producing iCHO cell lines described here comprise a powerful system and resource for further analysis of Wnt activity, including Wnt processing, secretion and posttranslational modification. These lines will help mitigate the current lack of adequate sources of most Wnt proteins, including those of human origin, and should expedite progress in critical research areas where Wnt function is either heavily implicated or poorly understood, such as regenerative medicine, stem cell biology, and cancer research. Moreover, the facile and robust method we developed to generate these cell lines will expedite generation of additional cell lines expressing other Wnts and their inhibitors to enable a deeper understanding of the mechanisms underlying this complex set of regulatory proteins. Furthermore, the low background expression and high inducibility of this system make it a molecular genetic platform technology attractive for the production of diverse classes of proteins, including those that are toxic when overexpressed, and those requiring association with the producing cell for their biologic activity. Therefore, this system has the potential to accelerate analysis of any signaling protein that is currently limited by protein production.

## Materials and Methods

### Molecular Biology

The following Wnt cDNAs were used in the Wnt expression vectors, most were obtained from OpenBiosystems: hWNT1 (accession number BC074799), hWNT3A (BC103921), hWNT5A (BC064694), hWNT7A (BC008811), hWNT11 (BC074791), hWNT16 (BC104945). All FLAG-tagged WNT proteins contain the hWNT3A signal sequence followed by a FLAG tag. A BspEI restriction site was used to join the FLAG tag with the remaining Wnt cDNA. Untagged Wnts were cloned into plasmid pJG011 (L3-pTRETight-eGFP-polyA-SV40-BSD-2L) using BamHI 5′ and either NotI or NheI 3′. pTRE-Tight was from Clontech.

### Cell Culture

Parental CHO cells were propagated in DMEM, 10% FBS, 0.4 mg/mL G418 (to maintain the rtTA transgene). Post-RMCE CHO cells were propagated in the above media plus 3 µg/mL Blasticidin and 5 ng/mL Doxycycline. 293A cells were co-transfected with the STF plasmid (gift from RT Moon) and the pcDNA3.1 His/lacZ plasmid (Life Technologies) in a 1∶6 ratio and selected with 1.2 mg/mL G418. LSL cells [43] were propagated in DMEM, 10% FBS. Super8XTOP-eGFP 293 Wnt reporter cells [44] were stably transfected with pTag-BFP-C (Evrogen) and selected with G418 (1.2 mg/ml). For co-culture imaging experiments, iCHO cells were seeded at 1000 cells per 10 cm plate and allowed to form colonies for 4 days prior to the addition of 3×10^6^ BSTG cells. 250 ng/ml Dox was added to plates after 24 hours to induce Wnt expression. Images were taken 48 hours later. iCHO colonies were located based on cell morphology and confirmed by corresponding gaps in the blue channel. Unprocessed GFP images taken with identical exposure settings are shown.

### RMCE

Parental CHO cells were transfected with PEI in a 6 well plate with 2 µg total of the incoming exchange plasmid and the Cre recombinase plasmid (pOG231) at a 2∶1 ratio. Media was changed after 6 hr. The following day, cells were trypsinized and expanded to 15 cm plates at low density. The following day, cells were treated with 2 µM Ganciclovir. Three days later media was replaced with fresh Ganciclovir-containing media. Four to seven days later, Gan-resistant colonies emerged and all cells on negative control plate were dead. At this point, Ganciclovir-containing media was replaced with media containing 3 µg/mL Blasticidin plus 5 ng/mL Dox (in our experience more colonies emerge with concomitant addition of Dox, possibly due to opening of the genomic locus where exchange occurs). True colonies emerged after four days of Blasticidin selection. Colonies were then picked and expanded.

### Western blotting and immunoprecipitation (IP)

Equal amounts of total protein from cell lysates and equal volumes of unconcentrated CM were separated by SDS PAGE using standard methods. Primary antibodies used were mouse anti-FLAG M2 (Sigma) and rabbit anti-FLAG (gift from Peter Gray, Salk). Secondary antibodies were conjugated to AF-680 (Life Technologies) or IRDye800 (Rockland) for scanning with LiCOR Odyssey. To prepare conditioned media (CM) for IP, 3.5×10^6^ CHO cells were seeded in a 10 cm dish in drug-free media plus 250 ng/mL Dox. 72 hours later, media was collected, filtered (0.2 µm) and stored at 4°C for less than one week before use. For IP, anti-FLAG sepharose (Sigma) was incubated overnight with CM, washed, resuspended in sample buffer with DTT, boiled, and used for SDS PAGE.

### Real-time bioluminescence monitoring assays

Twenty thousand 293A-STF cells were seeded into a 96 well white opaque plate (Corning) with or without twenty thousand engineered CHO cells in phenol red-free DMEM-F12 (Life Technologies), 10% serum, 100 µM D-Luciferin (Biosynth) and 250 ng/mL Dox. Real-time luminescence counts from three to six replicate wells were collected every 30 minutes by a temperature-controlled luminometer (Tecan M200) set to 37°C. While the background signal from parental CHO cells was relatively consistent from assay to assay, the level of induction was variable, depending on the reporter cells and plate format used. For this reason, and because the background readings are extremely low, we were unable to calculate a normalized induction for cross-comparison between experiments.

### Wnt purification

Wnt proteins were purified from 2–6 liters of iCHO CM. CM was complemented with 1% Triton X-100, 20 mM Tris-HCl pH 7.5 and 0.01% NaN_3_. The purification method consists of four consecutive steps performed on an Äkta Purifier (GE Healthcare) using the method previously described (Willert 2008). Wnt yields were determined by Coomassie staining of SDS-PA gels. Yields for WNT3A and 5A with and without FLAG tags averaged 40 ug/L.

### Flow cytometry

For surface Wnt analysis, iCHO cells were grown for 48 hours with 250 ng/mL Dox, harvested with EDTA and stained with anti-FLAG primary antibody (M2, Sigma) and anti-mouse secondary antibody AF568 (Life Technologies).

## Supporting Information

Figure S1 **Optimized RMCE selection strategy.**A) Schematic of positive and negative selection strategy to reduce the emergence of false-positive clones. B) Flow cytometry analysis of RMCE efficiency. Parental CHO cells (CHO111-134) were engineered by RMCE with a mCitrine expression cassette using the positive and negative selection strategy. Four pools of clones were analyzed, each of which had greater than 99% mCitrine positive cells.
(TIFF)

Figure S2 **Activity of hWNT5A purified from iCHO cells.**A) Coomassie stain of purified hWNT5A. B) 293-STF reporter assay showing that hWNT5A on its own doesn't activate STF, but is able to inhibit WNT3A activation of the pathway.
(TIFF)

Figure S3 **Flow cytometry analysis of surface FLAG-WNTs.**iCHO cells were induced with 250 ng/mL Dox and harvested after 48 hours with either 50 mM EDTA or 0.05% trypsin. They were then stained with anti-FLAG (M2) primary antibody and anti-mouse AF568 secondary antibody. Live cells (DAPI negative) are shown in the plots above. The top three panels are negative controls. iCHO WNT1 cells react strongly when harvested with EDTA, but the signal is decreased when the cells are harvested by trypsin.
(TIFF)

Figure S4 **WNT1 signals to neighboring cells.**Additional images from experiments as seen in Figure 4. Co-culture with A) parental iCHO cells, B) hWNT1-iCHO cells, C) hWNT3A-iCHO cells. Results were typical across three independent experiments.
(TIFF)

Figure S5 **SuperTOPeGFP expression is dose sensitive.**BSTG cells were treated for 48 hr with purified hWNT3A at the indicated concentrations, then harvested for flow cytometry analysis. The plots show the proportion and intensity of live GFP+ cells. The graph depicts the relative geometric mean intensity of GFP+ cells (P4 gate).
(TIFF)
