# Supplementary figures and images for: Use of a Molecular Genetic Platform Technology to Produce Human Wnt Proteins Reveals Distinct Local and Distal Signaling Abilities
Source: PLoS One. 2013 Mar 13;8(3):e58395. doi: 10.1371/journal.pone.0058395 (PMC3596392; doi:10.1371/journal.pone.0058395)

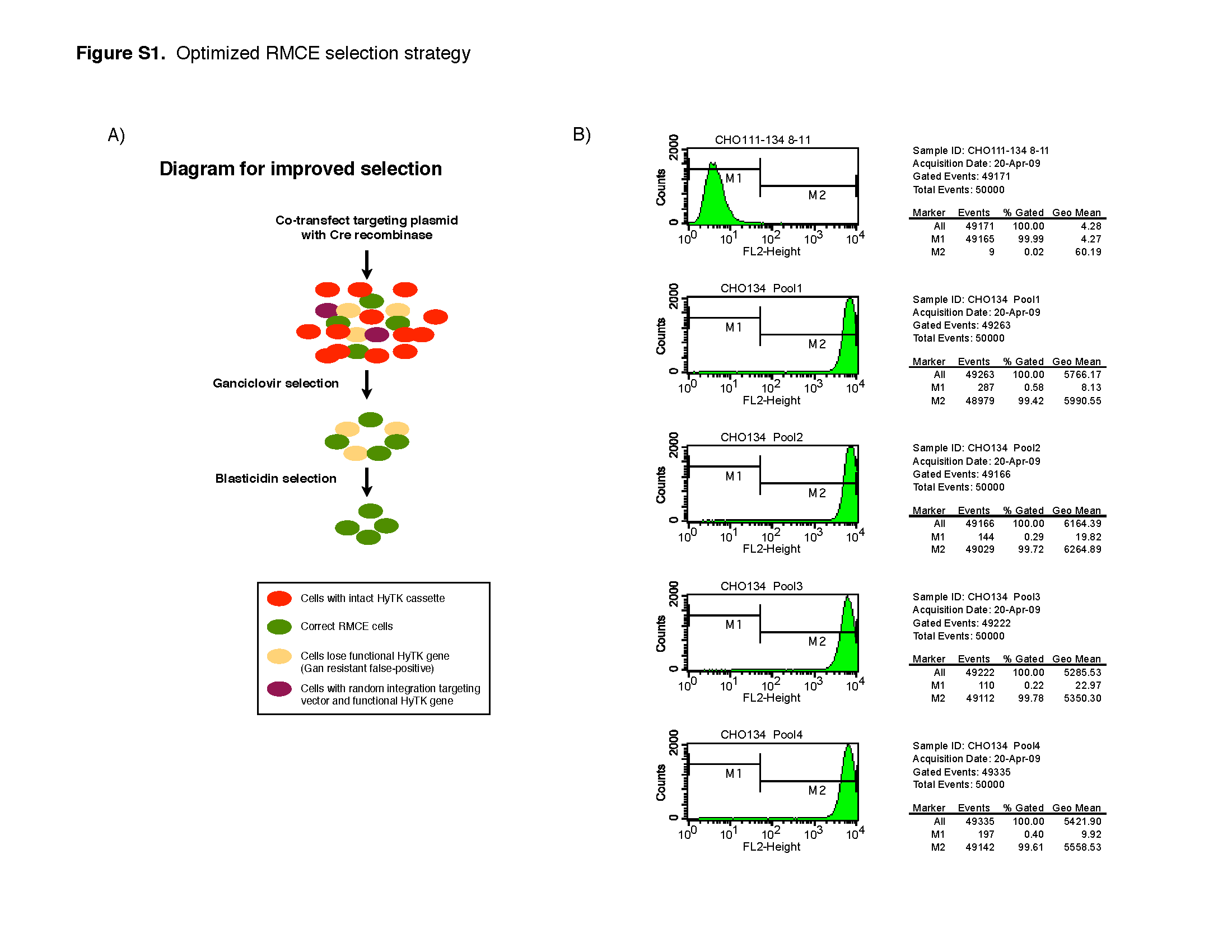

Supplement: Figure S1 — Optimized RMCE selection strategy.A) Schematic of positive and negative selection strategy to reduce the emergence of false-positive clones. B) Flow cytometry analysis of RMCE efficiency. Parental CHO cells (CHO111-134) were engineered by RMCE with a mCitrine expression cassette using the positive and negative selection strategy. Four pools of clones were analyzed, each of which had greater than 99% mCitrine positive cells. (TIFF) [file pone.0058395.s001.tiff]

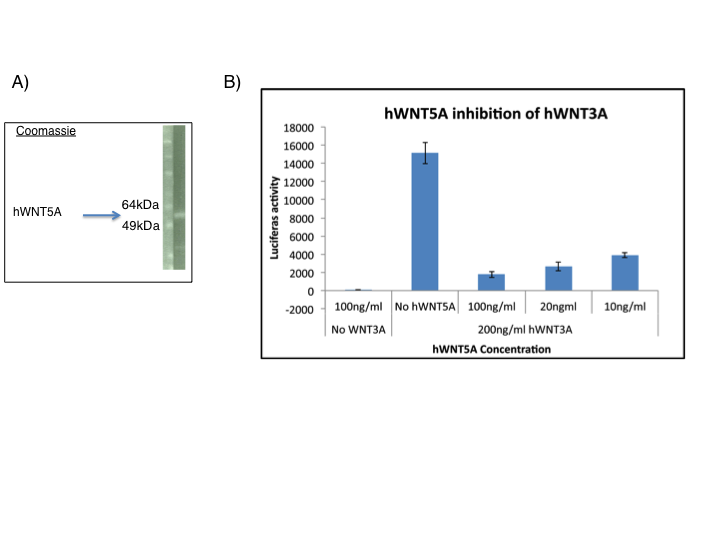

Supplement: Figure S2 — Activity of hWNT5A purified from iCHO cells.A) Coomassie stain of purified hWNT5A. B) 293-STF reporter assay showing that hWNT5A on its own doesn't activate STF, but is able to inhibit WNT3A activation of the pathway. (TIFF) [file pone.0058395.s002.tiff]

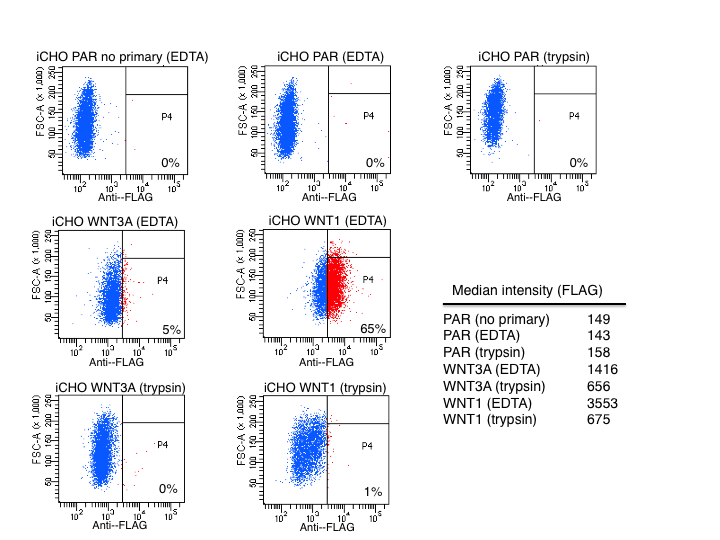

Supplement: Figure S3 — Flow cytometry analysis of surface FLAG-WNTs.iCHO cells were induced with 250 ng/mL Dox and harvested after 48 hours with either 50 mM EDTA or 0.05% trypsin. They were then stained with anti-FLAG (M2) primary antibody and anti-mouse AF568 secondary antibody. Live cells (DAPI negative) are shown in the plots above. The top three panels are negative controls. iCHO WNT1 cells react strongly when harvested with EDTA, but the signal is decreased when the cells are harvested by trypsin. (TIFF) [file pone.0058395.s003.tiff]

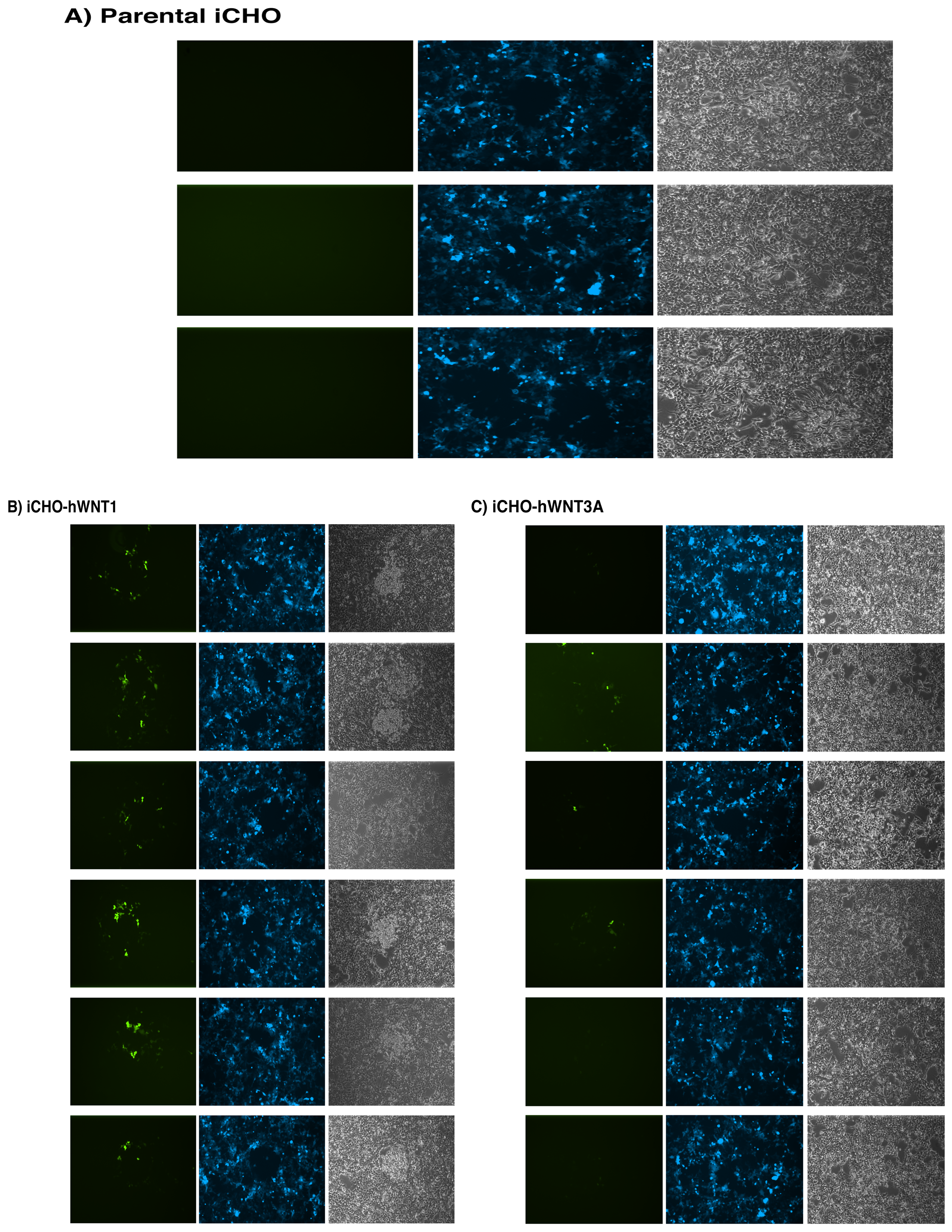

Supplement: Figure S4 — WNT1 signals to neighboring cells.Additional images from experiments as seen in Figure 4. Co-culture with A) parental iCHO cells, B) hWNT1-iCHO cells, C) hWNT3A-iCHO cells. Results were typical across three independent experiments. (TIFF) [file pone.0058395.s004.tiff]

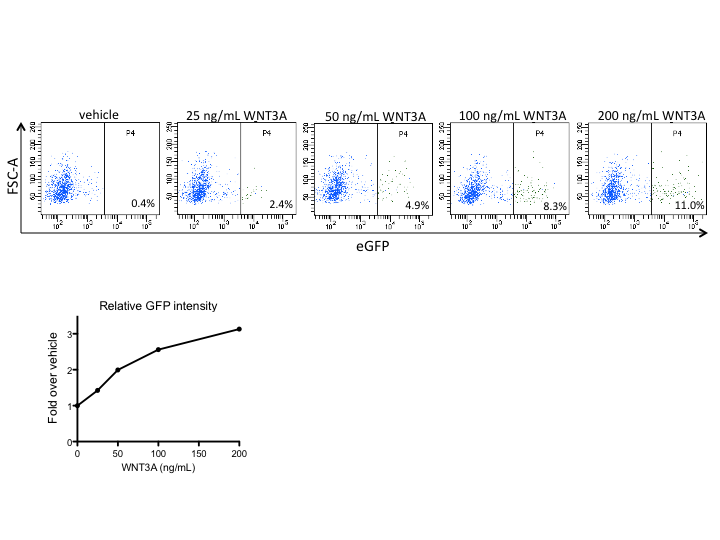

Supplement: Figure S5 — SuperTOPeGFP expression is dose sensitive.BSTG cells were treated for 48 hr with purified hWNT3A at the indicated concentrations, then harvested for flow cytometry analysis. The plots show the proportion and intensity of live GFP+ cells. The graph depicts the relative geometric mean intensity of GFP+ cells (P4 gate). (TIFF) [file pone.0058395.s005.tiff]
